# Agenesis of the right external iliac artery in a 23-year-old woman with left leg ischemia

**DOI:** 10.1016/j.radcr.2023.08.112

**Published:** 2023-09-23

**Authors:** Mahshid Bahrami, Ali Hajihashemi, Mahsa Geravandi

**Affiliations:** a Department of Radiology, Al-Zahra Hospital, Isfahan University of Medical Sciences, Isfahan, Iran; b Department of Radiology, Isfahan University of Medical Sciences, Isfahan, Iran

**Keywords:** Agenesis, External iliac artery, Ischemia

## Abstract

External iliac agenesis is an uncommon congenital issue characterized by the absence or incomplete development of the external iliac artery, a significant blood vessel supplying the lower limbs, potentially causing symptoms like pain, weakness, ischemia, and numbness. We are currently discussing a case of a 23-year-old woman who complained of pain in her left lower limb. A thorough work-up was conducted to rule out ischemia, and during the diagnostic process, a CT scan revealed the complete absence of the right external iliac artery. It can be concluded that this is a rare vascular anomaly that can lead to significant morbidity and mortality. Early diagnosis and prompt management are crucial for preventing complications such as limb ischemia and gangrene.

## Introduction

External iliac agenesis is a rare congenital condition, characterized by the absence or underdevelopment of the external iliac artery. This major blood vessel supplies blood to the body's lower limbs [1]. This condition can be unilateral or bilateral, leading to a range of symptoms, including pain, weakness, ischemia, and numbness in the affected limb. The exact cause of external iliac agenesis is not fully understood, but it is believed to result from abnormal development of the embryonic blood vessels during fetal development [2]. There is no cure for iliac agenesis, but treatment options may include medications to manage pain and improve blood flow and surgical interventions such as bypass grafting or angioplasty. It is essential for individuals with this condition to work closely with their healthcare providers to manage their symptoms and prevent complications [[3], [4]].

## Case presentation

A 23-year-old woman with no significant medical history presented to the emergency department complaining of left lower limb pain. She reported 1 month of left lower extremity pain that was progressively worsening. She explained that exercise exacerbated the condition, which also persisted at rest. One day before admission, she could not walk because of worsening calf pain. She denied any other GI or GU complaint, such as an inability to pass gas or any changes to her bowel or bladder function. There was no use of tobacco or alcohol. No history of prior trauma or instrumentation of the lower limb arteries was noted.

In the physical exam, the general appearance was normal. She was agitated but alert and oriented toward time, place, person, and situation. The heart and respiratory rates were 90 beats per minute and 19 breaths per minute, respectively. The temperature was 37.5°C, and the blood pressure was 140/100 mm Hg.

The abdominal examination was normal. The right lower limb neurological assessment (motor, sensory, and deep tendon reflexes) was intact. Its skin was warm with good turgor. The peripheral pulses of the right lower extremity were palpable.

The neurological assessment of the left lower leg was intact. Still, her skin was cold and cyanotic, and the peripheral pulses in the left dorsalis pedis, posterior tibialis, and popliteal arteries were not palpable. There was no evidence of asymmetry, muscular hypotrophy, or atrophy in both lower limbs.

At admission time, an examination of other systems showed no abnormalities.

**Fig. 1:**
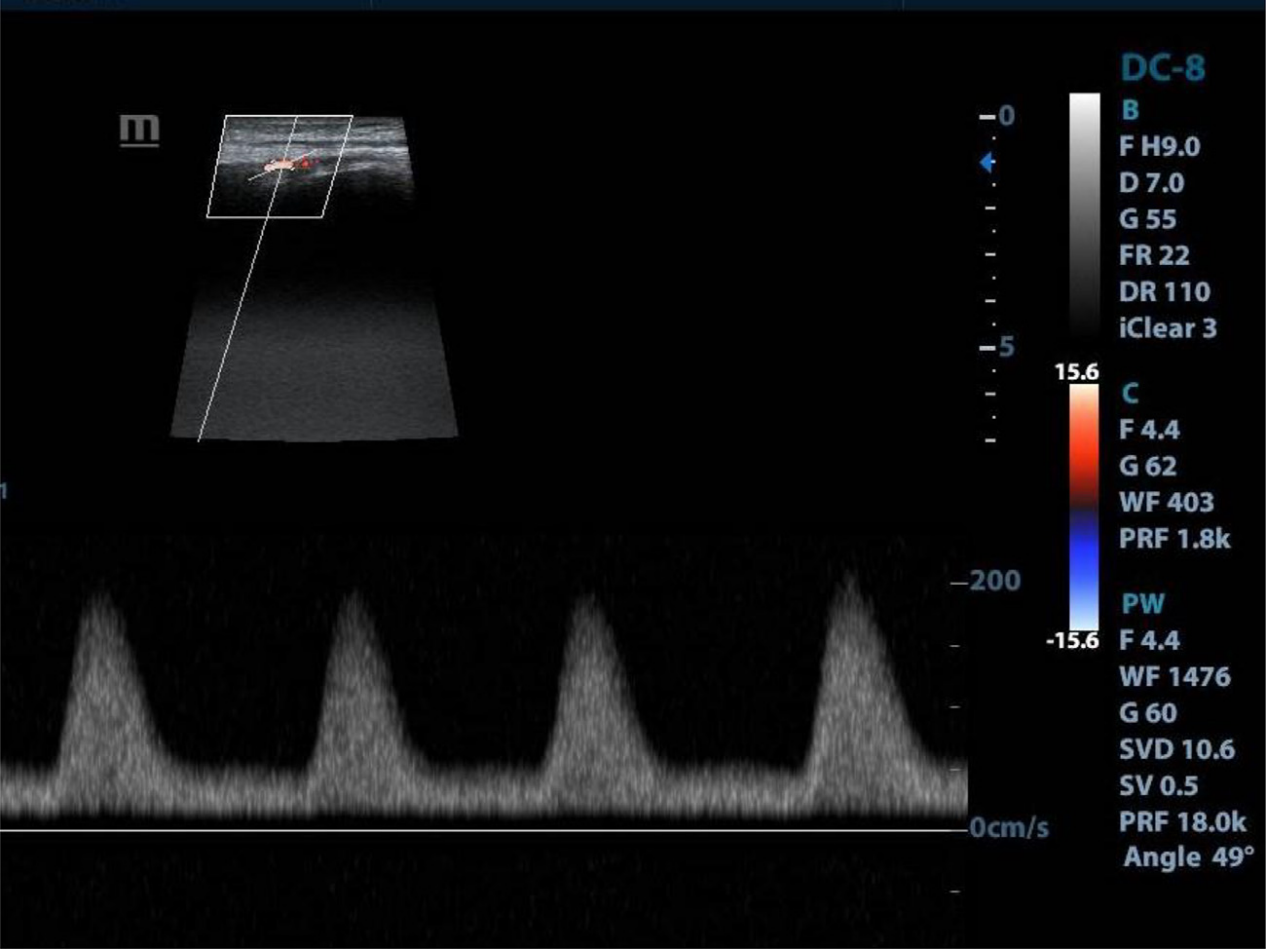
Doppler ultrasound of the left lower limb: There appears to be a monophasic waveform in the left superficial femoral artery.

Lab data revealed an erythrocyte sedimentation rate (ESR) of 30 mm/h and a C-reactive protein (CRP) of 50 mg/L. The complete blood count test findings were mild leukocytosis and normal red blood cell and platelet numbers. The blood urea nitrogen (BUN) and serum creatinine (Cr) were normal. Lab data showed no evidence of hypercoagulability, hyperlipidemia, or hepatorenal dysfunction. The Doppler ultrasound scan showed a monophasic waveform in the left superficial femoral artery. There was no flow in the anterior tibialis, peroneal, or posterior tibialis arteries (Fig. 1). Upon further questioning, she reported limitations to activities such as running or prolonged walking due to fatigue from 6 months ago.

**Fig. 2:**
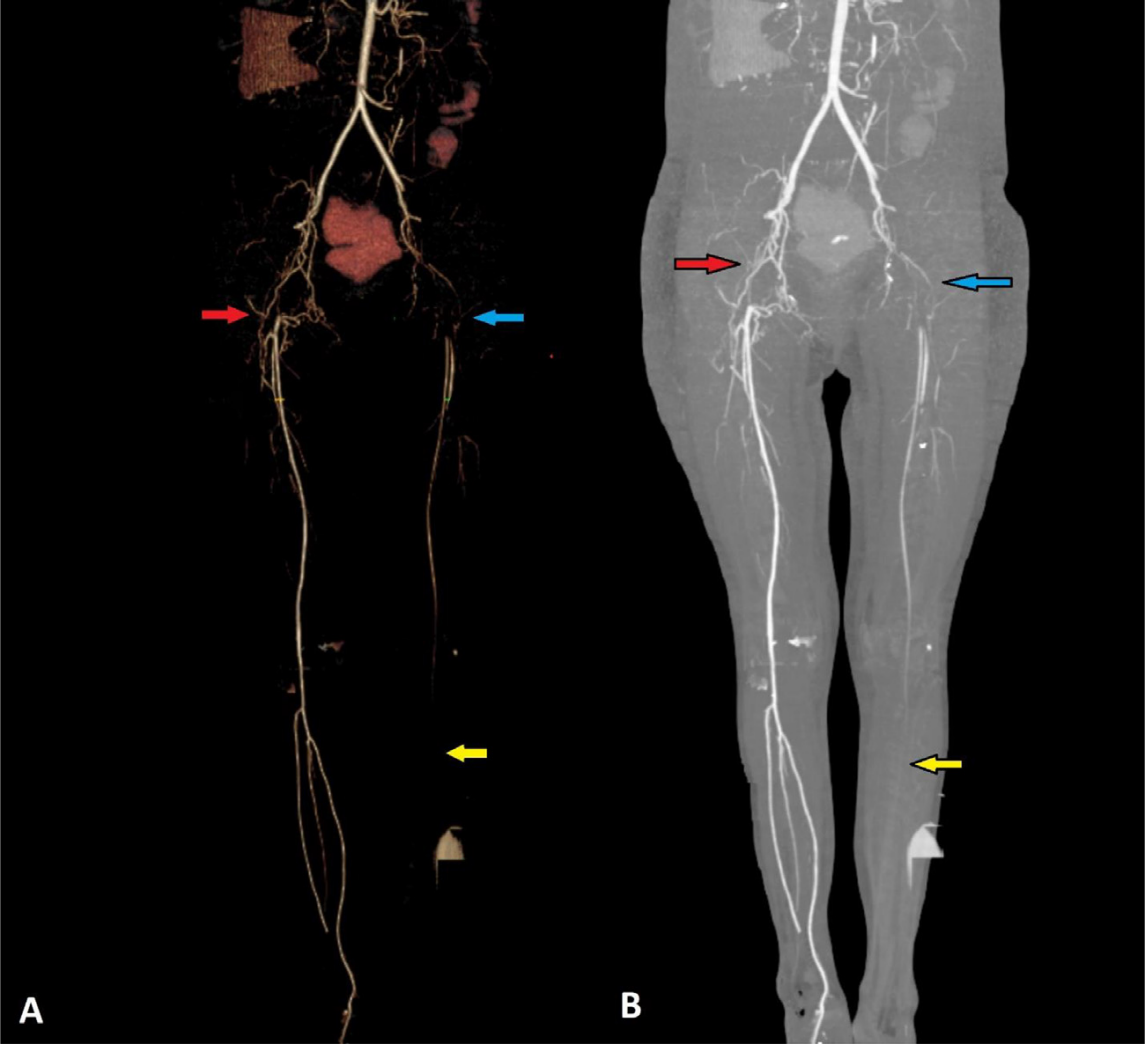
Lower limbs CT angiography, coronal view (A and B): Agenesis of the right external iliac artery led to opacification of multiple right-sided collateral vessels in the right internal iliac. (Red arrows) Complete thrombosis of the left external iliac artery (EIA) and common femoral artery (CFA). (Blue arrows) The superficial femoral artery (SFA) has decreased diameter without an obvious filling defect. The proximal parts of the anterior tibialis, peroneal, and posterior tibialis arteries have a filiform enhancement and are not opacified in other parts (Yellow arrows).

A CT angiogram was recommended due to the patient's physical exam and Doppler ultrasound. The CT angiogram showed a normal diameter and opacification of the left common iliac artery and its internal branches. Evidence of complete thrombosis of the external iliac artery (EIA) and common femoral artery (CFA) was noted. The superficial femoral artery (SFA) had decreased luminal diameter without thrombosis. The anterior tibialis, peroneal, and posterior tibialis arteries had filiform enhancement in the proximal part and were not opacified in other parts. The deep femoral artery (DFA) and popliteal artery were normal (Fig. 2).

**Fig. 3:**
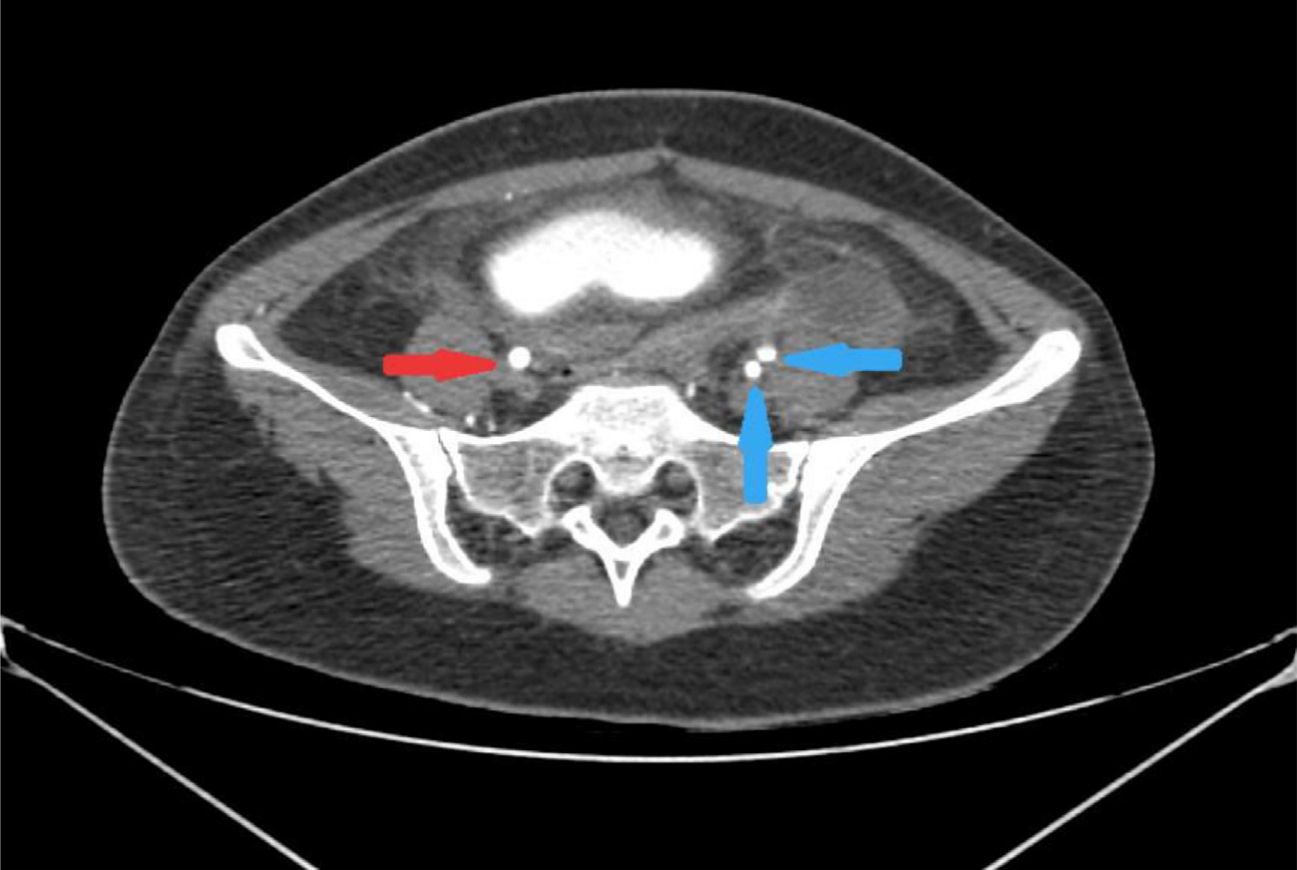
Enhanced abdominopelvic computed tomography (CT) scans; The external iliac artery on the right side was not visible (red arrow), while the left internal and external iliac arteries were visible (Blue arrow).

**Fig. 4:**
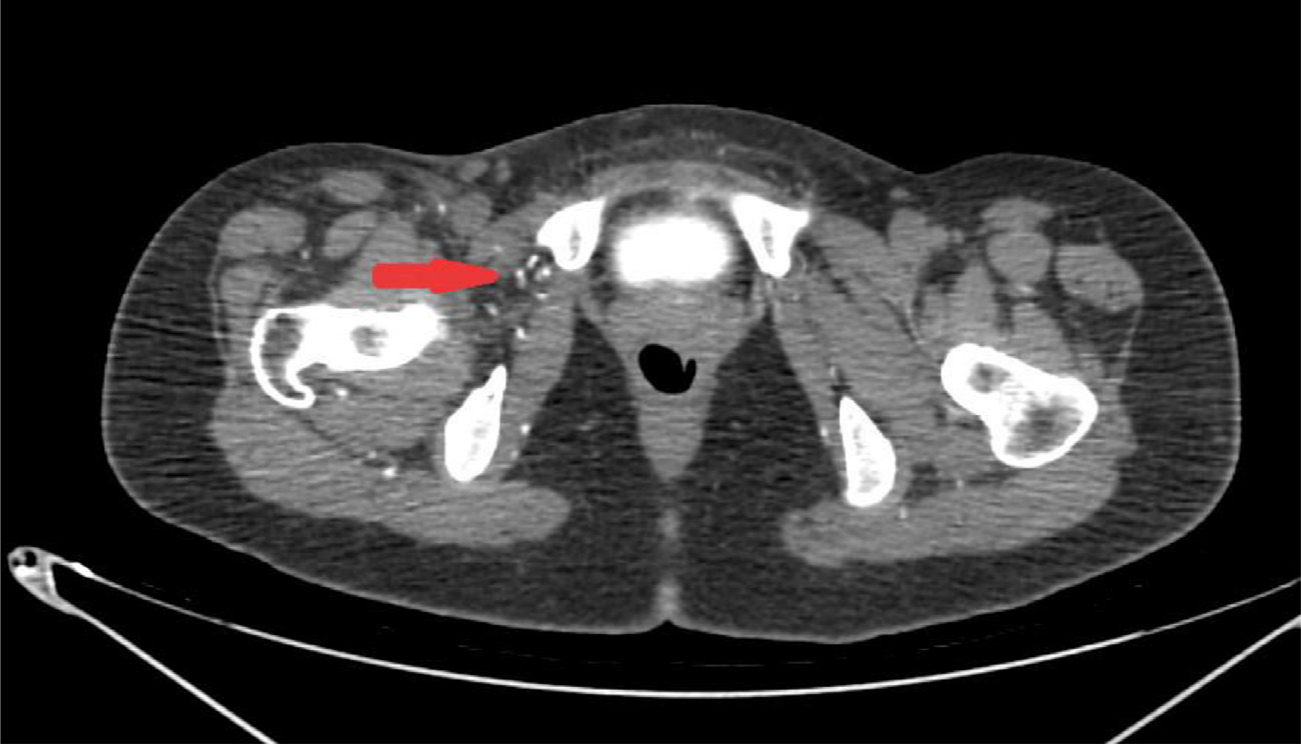
Axial abdominopelvic CT scan with contrast: There are several collateral vessels on the right side of the abdominal wall and extraperitoneal (arrow).

Incidentally, on the opposite side, the normal-appearing right common iliac artery ended abruptly at the point where the right external iliac artery would be expected to originate (Figs. 2 and 3). The proximal portion of the right external iliac artery lumen was not visualized, and there was no evidence of chronic thrombosis at the site of the right EIA (Fig. 3). The right CFA was opacified via multiple extraperitoneal and abdominal wall collateral vessels (Fig. 4).

Due to acute thrombosis of the left CFA, surgical embolectomy was considered. She was discharged without any obvious issues. One month later, follow-up showed no side effects at the surgical site, the cyanosis of the left limb disappeared, and pulses were detected. The patient underwent a successful surgical intervention using an iliofemoral graft, which restored blood flow to the right limb and improved her quality of life.

## Discussion

Congenital malformations of the iliofemoral arterial system are rare [5]. Greeb described only 6 cases in a series of about 8000 patients who underwent angiography of the pelvic artery [6]. Most reported cases have been iliofemoral arterial anomalies associated with persistent sciatic arteries [7,8].

Congenital malformation of the external iliac artery has been classified into 3 groups by Tamisier et al. [9]: group 1, anomalies in origin or course of the artery; group 2, hypoplasia or atresia compensated for by persistent sciatic artery; and group 3, isolated hypoplasia or atresia. Group 1 disorders are unlikely to cause chronic leg ischemia and are most often discovered at autopsy. A high incidence of aneurysm formation and arteriosclerosis of the sciatic artery associated with acute occlusion and embolization has been documented for group 2 disorders. Group 3 disorders are most likely suspected because of chronic leg ischemia.

In the current study, we present a rare case of right EIA agenesis that was referred to our hospital with signs of left leg ischemia. The CT angiogram showed a normal diameter and opacification of the left common iliac artery and its internal branches. However, coincidentally, on the opposite side, the normal-appearing right common iliac artery ended abruptly at the point where the right EIA would be expected to originate. Notably, individuals with ipsilateral isolated hypoplasia or atresia typically experience ischemia on the same side.

In nearly all patients, the congenital absence of the iliac arteries had been diagnosed incidentally, and many had no history of limb ischemic symptoms. This was eventually possible because the collateral vessels developed well and provided a good blood supply. Therefore, most patients were followed up without intervention [4].

In isolated hypoplasia or atresia of the external iliac artery (Tamisier group 3), chronic leg ischemia usually occurs in childhood to young adulthood, with a diagnosis between 12 and 44 years. Developed collateral vessels are found between the aorta and femoropopliteal system. Koyama et al. [10] described a patient with no episode suggestive of leg ischemia. They required no physical restriction for exercise such as running, climbing, or swimming until mild intermittent claudication developed 6 months before admission. Although this case is unlikely to have a group 3 disorder. In such cases, symptoms of leg ischemia may not always develop in young patients. Also, Doita et al. [2] presented a rare case of the congenital absence of the left common and external iliac arteries, with no limb ischemic symptoms or organ anomalies present. Although our patient had symptoms of ischemia of the left leg, the absence of EIA was observed on the right side of the patient, which has no special relationship with the ischemia of the opposite side.

In our case, the right CFA is opacified via multiple extraperitoneal and abdominal wall vessels. This finding helped us distinguish right external iliac artery agenesis from its thrombosis. An interesting thing to note is that patients with ipsilateral isolated hypoplasia or atresia usually have ischemia on the same side. However, our patient had ischemia on the opposite side, which had nothing to do with right external iliac agenesis. Also, in most of the reviewed studies, it was observed that there was a congenital common iliac artery along with EIA agenesis. Still, in our study, isolated agenesis of EIA occurred.

When an iliofemoral anomaly is observed, informing the patient and confirming whether other organ anomalies are present is essential. Ischemic symptoms will likely appear if the collateral circulation is damaged; thus, a careful preoperative assessment is required before surgery or catheter-based intervention. Moreover, surgical intervention such as a bypass procedure should be considered when limb ischemia occurs or if ischemic symptoms become progressively worse.

## Patient consent

Written informed consent for publication was obtained from the patient.
